# Commentary: Quantile treatment effect of zinc lozenges on common cold duration: A novel approach to analyze the effect of treatment on illness duration

**DOI:** 10.3389/fphar.2023.1152305

**Published:** 2023-04-06

**Authors:** Jonas Moss

**Affiliations:** BI Norwegian Business School, Oslo, Norway

**Keywords:** causal effect size, outcome measure, copulas, causality, potential outcomes, time-dependence

## Introduction

The effect of zinc lozenges on reducing the duration of the common cold is well-established (Hemilä, 2011), but it is not clear how different cold durations are affected by the lozenges. To be precise, define *T*_placebo_ and *T*_treatment_ as the length of a cold episode under placebo and treatment. We cannot observe both random variables at once, hence they are known as potential outcomes (Imbens and Rubin, 2015). The average treatment effect, defined as *ATE* = *E*(*T*_treatment_) − *E*(*T*_placebo_), can be estimate using randomized clinical trials. The investigation of Mossad et al. (1996) suggests that *ATE* ≈ − 4 days, hence zinc lozenge treatment reduces the average length of a cold episode by ∼4 days. However, the average treatment effect tells us little about the effect of the lozenges on cold episodes of a prescribed length, such as 3. Since the length cannot be less than 0, the average treatment effect cannot be directly applied in this case.

How can we quantify the effect of the zinc lozenges on the duration of a cold episode that would have had length *t* without treatment? Ideally, we would have liked to know the *conditional average treatment effect*

$$CATE\left(t\right)=E\left(T_{\text{treatment}}|T_{\text{placebo}}=t\right)-t.$$ (1)

But this and similar conditional quantities, such as conditional medians, are impossible to estimate from randomized clinical trials alone, as they depend on the joint distribution of *T*_placebo_ and *T*_treatment_. Estimation would require methods such as matching (Imbens and Rubin, 2015, chap. 18) coupled with severe statistical assumptions.

Hemilä et al. (2022) makes a case for using the *quantile treatment effect* (Doksum, 1974) when evaluating the effect of zinc lozenges on cold duration. The quantile treatment effect has been widely applied in economics, and certainly has its uses, especially in quantile regression (Koenker and Hallock, 2001). However, Hemilä et al. (2022) claim to estimate a quantity similar to *CATE*(*t*) using the quantile treatment effect. For they write, along with numerous similar claims, that

[…] the [quantile treatment effect] analysis indicates that 15- to 17-day colds were shortened by 8 days, and 2-day colds by just 1 day, for the group taking zinc lozenges.

This conclusion is too strong and potentially misleading, as the quantile treatment effect only indicates anything of the sort when quite stringent assumptions on the joint distribution of (*T*_treatment_, *T*_placebo_) are met.

### The quantile treatment effect

The quantile treatment effect at quantile *p* is defined as

$$QET\left(p\right)=Q_{\text{treatment}}\left(p\right)-Q_{\text{placebo}}\left(p\right),$$ (2)

where *Q*_treatment_ and *Q*_placebo_ are the quantile functions for the outcome under treatment and placebo.

The authors reached the conclusion cited above by substituting *p* for *F*_placebo_(*t*) (the distribution function of the cold duration under placebo) in the equation for the quantile treatment effect. This substitution yields

$$\phi \left(t\right)=Q_{\text{treatment}}\left(F_{\text{placebo}}\left(t\right)\right)-t.$$ (3)

Under the assumption that there is a deterministic and increasing relationship between *T*_placebo_ and *T*_treatment_, it is easy to show that *ϕ*(*t*) = *CATE*(*t*). However, a deterministic relationship between *T*_placebo_ and *T*_treatment_ is highly unlikely. To see why, consider two patients with exactly the same cold duration, one who is 58 and male and one who is 17 and female. If the relationship between placebo outcome and treatment outcome is deterministic, both patients must have *exactly the same* cold duration when treated with zinc lozenge. This assumption is virtually guaranteed to be false.

There are infinitely many possible conditional average treatment effects *CATE*(*t*) compatible with the observed marginal data only. A way to generate some of them is to use a Gaussian copula (Nelsen, 2007), modelling the dependence structure of a bivariate normal. We can parameterize the conditional average treatment effect by *ρ*, obtaining a family of functions *CATE*(*t*; *ρ*). It is easy to show that *CATE*(*t*; 1) = *ϕ*(*t*), that *CATE*(*t*; 0) = *E*(*T*_treatment_) − *t*, and that *CATE*(*t*; −1) = *Q*_treatment_ (1 − *F*_placebo_(*t*)) − *t*.

**FIGURE 1:**
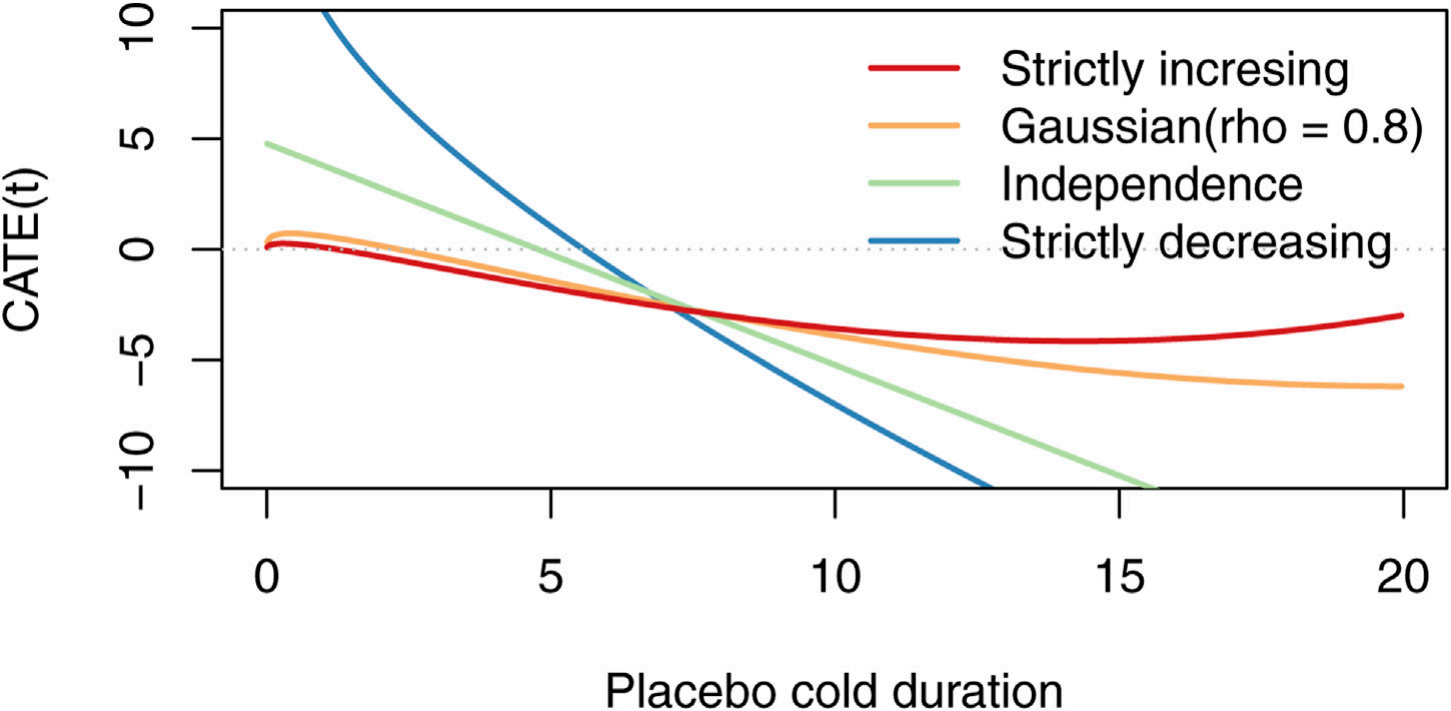
Possible CATE curves for the zinc data set when the correlation of the Gaussian copula varies. Hemilä et al. (2022), (Figure 2(B)) based their analysis on a line similar to the “strictly increasing” line, but used non-parameteric estimators for the quantile functions instead of maximum likelihood for gamma and Weibull.

Figure 1 displays some of the possible conditional average treatment effect curves for the data of Petrus et al. (1998), Prasad et al. (2000), and Prasad et al. (2008) [found in the supplementary materials of Hemilä et al. (2022)] when the copula is Gaussian. For computational convenience, we have assumed that the placebo group is gamma distributed and the treatment group is Weibull distributed. We estimated their parameters using maximum likelihood, and calculated the *CATE*(*t*) curves using numerical integration. As can be seen, there are conditional average effect curves of many shapes. The corresponding plot of Hemilä et al. (2022) is Figure 2B, where they used non-parametric estimators for *Q*_treatment_ and *Q*_placebo_, and the *x*-axis is on the percent scale instead of the outcome scale.

## Conclusion

Comments similar to mine have been made in the context of economics by, e.g., Abadie et al. (1998) and Koenker and Bilias (2002), who, in our terminology, emphasize that the quantile treatment effect cannot be used to estimate *CATE*(*t*), but that it still have its uses.

It is important to understand how illness duration without treatment relates to illness duration under treatment. This could be done using conditional average treatment effect. It is, however, important not to use methods that cannot answer such questions in a rigorous way, as is the case with the quantile treatment effect suggested by Hemilä et al. (2022).
